# Highly Efficient Arsenic Removal Using a Composite of Ultrafine Magnetite Nanoparticles Interlinked by Silane Coupling Agents

**DOI:** 10.3390/ijerph9103711

**Published:** 2012-10-16

**Authors:** Shu-Chi Chang, Yu-Han Yu, Cheng-Hao Li, Chin-Ching Wu, Hao-Yun Lei

**Affiliations:** 1 Department of Environmental Engineering, National Chung Hsing University, 250 Kuo-Kuang Road, Taichung 40227, Taiwan; Email: hunter730502@yahoo.com.tw (Y.-H.Y.); s19886100@hotmail.com (C.-H.L.); tmac770726@hotmail.com (H.-Y.L.); 2 Department of Public Health, China Medical University, 91 Hsueh-Shih Road, Taichung 40402, Taiwan; Email: ccwu4710@yahoo.com.tw

**Keywords:** arsenic, adsorption, magnetite, nanoparticle, magnetite composite

## Abstract

Arsenic (As) contamination in groundwater is a great environmental health concern and is often the result of contact between groundwater and arsenic-containing rocks or sediments and from variation of pH and redox potentials in the subsurface. In the past decade, magnetite nanoparticles (MNPs) have been shown to have high adsorption activity towards As. Alerted by the reported cytotoxicity of 5–12 nm MNP, we studied the adsorption behavior of 1.15 nm MNP and a MNP composite (MNPC), MNPs interlinked by silane coupling agents. With an initial concentration of As at 25 mg L^−1^, MNPs exhibited high adsorption capacity for As(V) and As (III), 206.9 mg·g^−1^ and 168.6 mg·g^−1^ under anaerobic conditions, respectively, and 109.9 mg·g^−1^ and 108.6 mg·g^−1^ under aerobic conditions, respectively. Under aerobic conditions, MNPC achieved even higher adsorption capacity than MNP, 165.1 mg·g^−1^ on As(V) and 157.9 mg·g^−1^ on As(III). For As(V) at 50 mg L^−1^, MNPC achieved an adsorption capacity as high as 341.8 mg·g^−1^, the highest in the literature. A kinetic study indicated that this adsorption reaction can reach equilibrium within 15 min and the rate constant of As(V) is about 1.9 times higher than that of As(III). These results suggested that MNPC can serve as a highly effective adsorbent for fast removal of As.

## 1. Introduction

Arsenic (As) is regarded as one of the most toxic heavy metals and has ranked at the top for more than a decade on the Priority List of Hazardous Substances according to Comprehensive Environmental Response, Compensation, and Liability Act (CERCLA) in the USA. Even now, tens of millions of people are still suffering from exposure to drinking water with high arsenic concentrations. In Bangladesh alone, there are about some 50,000,000 people at risk exposed to As-contaminated water [1].

Arsenic poisoning can be acute or chronic. While acute poisoning is usually due to mistaken ingestion of foods or water with high As concentrations, chronic poisoning is often caused by long term exposure to low-level As from contaminated foods, water, and air. The endpoints of arsenic toxicity could be carcinogenicity (cancers in various organs, especially in skin, lungs, and bladder), systemic toxicity, (e.g., internal organ damage such as respiratory, circulatory, and renal), reproductive toxicity (neural tube defects), and neurotoxicity (brain cell damage) [2,3,4,5,6]. Therefore, there is no doubt that effective removal of medium to low level of As in daily drinking water is essential to prevent chronic arsenic poisoning. Since a person only consumes about 2 liters of water for drinking but may consume more than 200 liters every day for other purposes, effective removal of As from drinking water at point of use is more relevant and is in urgent demand. 

Other than traditional As-removal technologies, like coagulation and flocculation, sedimentation and filtration, ion exchange [7], reverse osmosis [8], membrane filtration [9], and adsorption [10], recently magnetite nanoparticles (MNPs) have emerged as a viable alternative due to their high adsorption capacity [11,12,13]. MNPs smaller than 16 nm will exhibit unique superparamagnetic properties [14] allowing them to be dispersed in water. Thus, they are suitable for completely mixed reactor applications, such as coagulation followed by sedimentation. This approach may have advantages over membrane filtration or fixed bed filtration due to the absence of fouling or plugging problems [13]. However, they may encounter difficulties in recovery because of their smaller size and electric charge on the surface, rendering their suspensions highly stable. Furthermore, iron oxide nanoparticles with 5–12 nm diameters have been reported to possess cytotoxicity associated with the reduced viability of rat neurons, increased cytoskeletal disruption, and neurite growth disruption [15]. Ultrasmall superparamagnetic iron oxide (USPIO) particles between 10 and 100 nm are optimal for intravenous administration as a contrast agent in magnetic resonance imaging, but iron oxide nanoparticles smaller than 10 nm are not suitable for this application because they are readily removed through renal clearance [16]. Therefore, we prepared both MNPs much smaller than 4.0 nm and MNPC of a much larger size (usually larger than 100 nm). Their respective adsorption capacities for As(III) and As(V) were investigated and the recovery of these two adsorbents from treated water were also evaluated. A further kinetic experiment was performed for MNPs at environmentally-relevant levels of As to determine the feasibility of fast removal in drinking water.

## 2. Results and Discussion

### 2.1. Results

#### 2.1.1. Synthesis and characterization of MNPs and MNPCs

**Figure 1:**
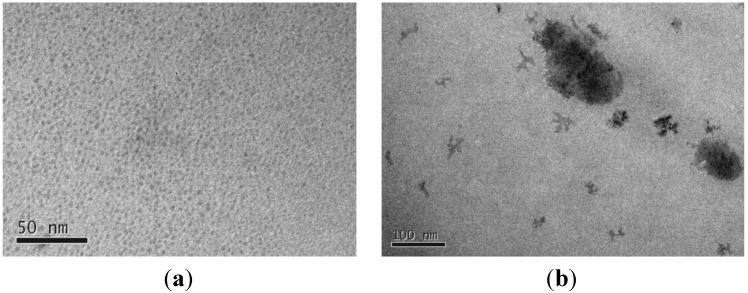
TEM micrograph of MNP (**a**) and MNPC (**b**). The bars in panels (**a**) and (**b**) represent 50 nm and 100 nm, respectively.

In this study, we successfully modified a coprecipitation method to produce hundreds of grams of sub-4 nm MNPs per batch. The particle shape and size were observed using a transmission electron microscope (TEM) as shown in Figure 1(a). Due to the limited resolution, an exact image cannot be effectively displayed in the micrograph. Here, the electron image showed that the size of individual nanoparticles and the aggregates of these MNPs were 1.15 nm and 3.02 ± 0.32 nm (mean ± CI 95%), respectively. Figure 1(b) shows that the MNPCs prepared in this study are not uniform and some of them have tree-like structures. X-ray diffraction measurements have been performed twice, before and after the adsorption experiments. Both of them clearly matched the characteristic magnetite crystal peaks according to the Powder Diffraction Files of the International Centre for Diffraction Data. The specific surface area (SSA) has been measured twice and the average was 65.8 m^2^·g^−1^. This value is much less than the calculated value of 3.02 nm and 1.15 nm aggregates, *i.e.*, about 386 m^2^·g^−1^ and 1,241 m^2^·g^−1^. This lower SSA measurement may be caused by further aggregation of the nanoparticles upon drying because BET measurement requires a dried sample.

After the sol-gel synthesis, MNPC has a high water content. After drying, its color turned from dark green to dark greenish brown indicating some of the MNP in the composite may have been oxidized to form maghemite or hematite since the synthesis reaction is performed under aerobic conditions. The aggregate size of dried MNPC ranged from 3 to 8 mm. This composite had been ground to fine granules and then observed under a TEM. The grain sizes varied and MNPs were embedded on the surface of the polyethoxysilane matrix (Figure 1(b)).

#### 2.1.2. As Adsorption by MNPs under Anaerobic and Aerobic Conditions

**Figure 2:**
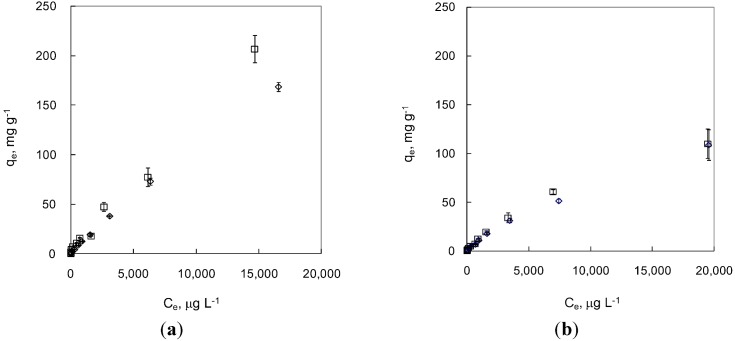
Adsorption of As(III) and As(V) by MNP under anaerobic (**a**) and aerobic (**b**) conditions. Empty rhombus represents As (III) and empty square represents As(V). Error bars indicate CI 95%.

The results of the adsorption experiments using MNPs were very different for As(III) and As(V) under anaerobic and aerobic conditions (Figure 2). In this study, under anaerobic condition (simulating groundwater in an undisturbed deep aquifer), the adsorption capacity of As(V) on MNPs is as high as 206.9 mg·g^−1^, which is significantly higher than that seen for As(III) (168.8 mg·g^−1^). To the authors’ knowledge, this adsorption capacity for As(V) is the highest reported in the literature for magnetite nanoparticles.

Under aerobic conditions (simulating aerated surface water or groundwater from a shallow aquifer), the adsorption capacities of MNPs were only significantly different for As(III) and As(V) in the concentration range of 10–1,000 μg·L^−1^where As(V) sorbed more onto MNP than As(III). Above this concentration range, the difference is minimal. Furthermore, the adsorption capacity under aerobic conditions is significantly (about 36–47%) lower than that under anaerobic conditions. This trend is expected because magnetite nanoparticles can be easily oxidized to form maghemite and hematite upon exposure to oxygen and these two iron oxides have much lower adsorption capacity than magnetite. 

#### 2.1.3. Kinetic Study of MNPs under Anaerobic Conditions

**Figure 3:**
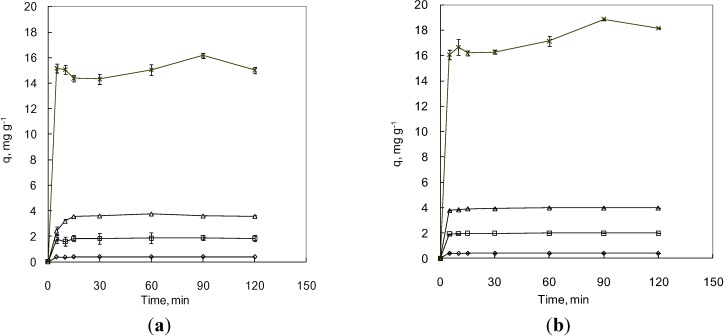
Adsorption of As(III) (**a**) and As(V) (**b**) by MNP under anaerobic condition. Rhombus, square, triangle, and cross represent initial concentrations of 10, 50, 100, and 500 μg·L^−^^1^, respectively. Error bars indicate CI 95%.

To determine if MNPs can serve as an effective adsorbent for As under more environmentally-relevant conditions, *i.e.*, As concentration ranged of 10–500 μg·L^−^^1^, pH 8.1, and anaerobic conditions, a kinetics study was performed and the results are shown in Figure 3. One can tell that most of the adsorption reactions reach equilibrium within 5 min. A pseudo-first order model is applied [17] to estimate the rate constants for As levels lower or equal to 100 μg·L^−^^1^. The rate constants for As(III) and As(V) are 12.32 ± 1.00 h^−1^ (mean ± CI 95%) and 23.13 ± 3.12 h^−1^ (mean ± CI 95%), respectively (Table 2). The coefficients of determination (R^2^) are not close to 1 due largely to the fact few data points are available before reaching equilibrium. Thus, these rate constants may be underestimated. Data points cannot be obtained at earlier times (less than 5 min) because the test resume bottles had to undergo magnetic separation for at least 3 min before sampling the supernatant. 

#### 2.1.4. As Adsorption by MNPCs under Aerobic Conditions

**Figure 4:**
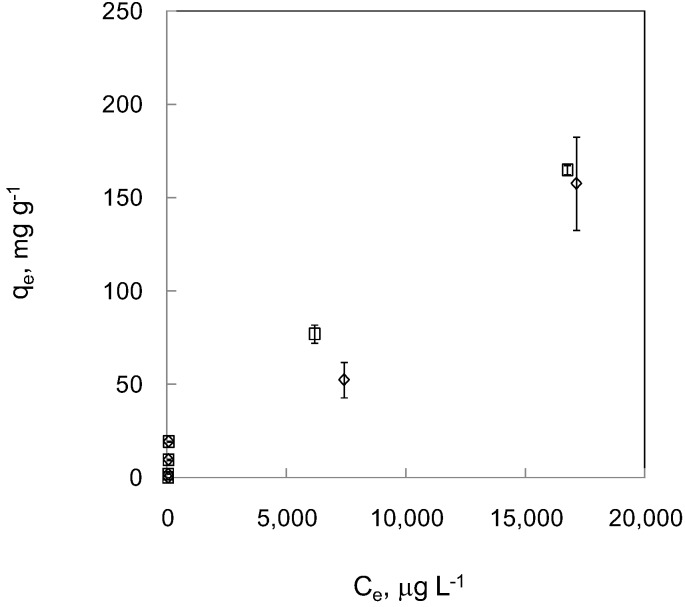
Adsorption of As(III) and As (V) by MNPC under aerobic condition. Empty rhombus represents As (III) and empty square represents As(V). Error bars indicate CI 95%.

For MNPCs, adsorption experiments were only performed under aerobic conditions since MNPCs are prepared in an aerobic environment. The adsorption capacities were even higher than those of MNP (Figure 4). At lower concentration ranges, 100 to 5,000 μg·L^−1^, the removal of As(III) and As(V) is 91.7–97.7 % and 89.6–97.4 %, respectively. For higher concentration ranges, 5,000 to 25,000 μg·L^−^^1^, the removal rates dropped to 33.0–38.6%. Even so, these removal rates are still higher than those of MNPs under aerobic conditions. The variation of these adsorption data is significantly larger than those of MNPs.

### 2.2. Discussion

For MNP synthesis, two different methods were tried, *i.e.*, microemulsion and coprecipitation [18,19]. According to our experience, the coprecipitation method is much easier in operation and the cost is about 95% less than microemulsion method on bench scale. Heating in a temperature-controllable sonication bath not only resulted in more monodisperse and smaller particle sizes but also increased the production up to hundreds of grams per batch. Unlike the documented coprecipitation method requiring high ionic strength [18], our method can readily make MNPs with a mean aggregate diameter of 3.02 nm at low ionic strength. This smaller size renders the nanoparticles superparamagnetic that allows the particles to disperse themselves in liquid, which is very favorable in adsorption applications [12,14]. From the TEM micrograph in Figure 1a, one can see the uniform size distribution of the resulting nanoparticles. After a mild probe sonication, the shape of the nanoparticles cannot be clearly imaged under a 120 KeV TEM. Since probe sonication usually breaks the weak chemical bonds such as hydrogen bonds, ionic bonds, and hydrophobic interactions [20], the actual size of the particles may be less than 1.0 nm. Dynamic light scattering measurement was also performed, but no consistent results could be obtained. Such measurement discrepancies has been reported for MNPs by other researchers [21]. 

Among all tests, under anaerobic conditions, the highest adsorption capacity of MNPs on As(III) and As(V) are 168.8 mg·g^−1^and 206.9 mg·g^−1^, respectively, The adsorption capacity on As(V) are the highest in the literature for magnetite [11,13,14,22] (Table 1). Comparing to the reported adsorption capacity of iron oxide nanoparticles with sizes less than 20 nm at pH values around 8.0, the MNPs in this study appeared to have higher adsorption capacity than larger particles. Even though the measured SSA of the MNPs in this study was not as high as other nanoparticles of similar or larger sizes, their adsorption capacity appeared to have no negative impact. One plausible reason is that the actual SSA may differ from that measured on a BET instrument. The dried aggregates in the solid phase used for the measurement may have smaller SSA than the dispersed nanoparticles in the liquid for adsorption reactions. A simple calculation could be done by using the measured SSA and the resultant site densities are in the range of 13.3 and 25.2 sites nm^−^^2^, which is not reasonable when comparing with the recently published data based on the analysis by extended X-ray absorption spectroscopy (EXAFS) [11,23,24,25]. According to the EXAFS analysis, the theoretical maximum site density is eight sites per square nanometer on the [111] facet of magnetite or maghemite nanocrystals. This site density is much lower than the value calculated by using the sorbed As on measured SSA in this study. If all facets were considered and the probability for the defects existence on different facets is taken into account, the average site density will be less than two sites per square nanometer [11]. Therefore, the BET measurement probably has underestimated by at least 6.65 to 12.6 times the SSA of the MNPs synthesized in this study. In other words, the actual SSA of MNPs should be at least 438 m^2^·g^−^^1^. Another reason for the high adsorption capacity of ultrafine MNPs than that of 20-nm or larger MNPs is the unique superparamagnetism property of MNPs smaller than 16 nm [14] that allows them to be dispersed in water instead of forming chain-like structures or other aggregation forms. Thus, MNPs smaller than this critical dimension, 16 nm, are expected to have much higher adsorption capacity. 

The reasons why MNPs can adsorb more As(V) than As(III) under anaerobic conditions but not so under aerobic conditions are discussed below. The major adsorption mechanisms of MNP have been suggested to be electrostatic interaction, surface complexation and site density. In this study, considering the fixed pH, the same adsorbent (similar MNPs), the dominant species of arsenite (H_3_AsO_3_^(^^0^^)^)and arsenate (H_3_AsO_4_^2^^−^), and the negatively charge surface of MNPs, the most likely dominant mechanism is surface complexation and the density of specific sites. For surface complexation, the major binding is that the pyramid of As(V)O_4_ forms ^2^C bidentate bonding or forms outer-sphere complex [23]. For As(III)O_3_, the major complexation is by forming hexanuclear ^3^C tridentate bonding [24,25]. This is very different from other iron oxides, such as hydrous ferric oxide and goethite [26]. Thus, the binding of As(V)O_4_ on MNPs is more versatile and allows higher surface coverage while the binding of As(III)O_3_ is dominated by one type that requires surface vacant sites. This is why As(V) could be adsorbed more on MNPs than As(III) under anaerobic conditions. But this discriminative adsorption behavior will change once the environment turns aerobic. This is because magnetite (Fe_3_O_4_) can easily be oxidized to form maghemite (γ-Fe_2_O_3_) at room temperature and hematite (α-Fe_2_O_3_) at much higher temperature [27]. Maghemite nanoparticles of sizes ranging from 3.8 to 18.6 nm have been tested for As(V) adsorption and the results showed the maximum adsorption capacity is lower than 25 mg·g^−^^1^ at pH 7.0 and pH 9.0 [22]. Another study on As(III) adsorption on 6-nm maghemite at pH 7.0 showed comparable results as 11-nm magnetite at pH 8.0 indicating that the negative impact of MNP oxidation on As(III) adsorption is much less than that on As(V) [11]. Thus, it is in good agreement with the literatures that the adsorption capacities are lower for both As(III) and As(V) under aerobic conditions than those under anaerobic conditions. The transformation of magnetite to maghemite has a higher negative impact on As(V) than on As(III).

**Table 1:** Comparison of the adsorption capacity of different iron oxide on As(III) and As (V).

Adsorbent	Size (nm)	pH	SSA (m^2^·g^−1^)	q_e_, max				References
				A(III) (mg·g^−1^)	As(V) (mg·g^−1^)	As(III) (nm^−2^)	As(V) (nm^−2^)	
MNP	3.02 ± 0.32	8.0	65.8	168.8	206.9	-	-	This study
				108.6	138.1	-	-	This study
MNPC	-	8.0	-	157.9	165.1	-	-	This study
Maghemite	6	7	174	172.5	-	8.1	-	[11]
Magnetite	12	8.0	-	-	~200 **	-	-	[14]
Magnetite	11.72	8.0	98.8 *	114.9	172.5	9.3 *	14.0 *	[13]
	20	8.0	60	29.2	5.9	3.8 *	0.8 *	
	300	6.1	3.7	1.5	0.75	3.3 *	1.6 *	
Maghemite	3.8 ± 0.8	7.0	203.2	-	20.0	-	0.04	[21]
		9.0			12.5	-	0.02	

* Calculated by Yean *et al.* [13]. ** Estimated by the authors.

For the kinetic study, since all the samples must be placed upon a rare earth magnet for at least 3 min before taking the supernatant, the data collected at 5 min only allowed the dispersed MNPs to fully react with As in aqueous phase for about 2 min. Thus, the estimations of rate constants in this study are rather conservative (Table 2). The rate constants of MNP adsorption in this study are about 11.0–13.4 times higher than those of the 20-nm MNP at 100 μg·L^−^^1^ of As concentration [12]. Our result is in good agreement with other researchers who indicated that the rate constant of As(V) adsorption is about 1.54 times higher than that of As(III) at low dosage at 0.05g L^−^^1^ [12]. Apart from the 3-min settling of MNPs on a magnet, the residual MNPs in the supernatant can also result in a slight underestimation of the rate constants. Therefore, these MNPs are highly suitable for point-of-use applications only if they can be effectively recovered or immobilized.

**Table 2:** Adsorption rate constants of magnetite nanoparticle at different initial concentrations

Initial concentration (μg·L^−1^)	K_ads_ in min^−1^ (R^2^)	
	As(III)	As(V)
10	0.222 (0.768)	0.422 (0.677)
50	0.201 (0.721)	0.400 (0.871)
100	0.193 (0.999)	0.334 (0.839)

From the adsorption experiment on MNPCs, there is no significant difference between As(V) and As(III) except at the concentration at 10,000 μg·L^−1^. A similar phenomenon is also observed in MNP adsorption experiments under aerobic conditions. However, on average, MNPCs exhibited 1.48 times higher adsorption capacity than MNPs, supporting the feasibility of increasing the surface area through embedding MNPs on the polyethoxysilane matrix. Furthermore, interestingly, MNPC grains seemed to retain the superparamagnetic property showing no attraction between each other, but strong attraction to a rare earth magnet. To test the stability of MNPCs under acidic conditions, MNPCs were immersed into an acidic solution at pH 1.0. No observable change in color, shape, and size of the composite, even after a 40-day immersion, was noted. In the past, smaller MNPs have been criticized for their quick dissolution at pH values of less than 4.0. Our MNPCs may have good applications in the lower pH range.

The composite-type approach has been tried in other forms by researchers, such as iron coated sands [28], and iron oxide on activated carbons [29]. Here, we tried to synthesize a three dimensional tree-like silica structure embedded with MNPs. Nanometer-scale branch- and twig-like structures were observed, indicating successful synthesis (Figure 1(b)). By comparing the reported adsorption capacity of 300 nm magnetite particles on As(III) at pH 8.0 [13], the adsorption capacity of MNPCs in this study is 99 times higher. The adsorption capacity on As(V) is expected to be at least 220 times higher than the reported 300 nm magnetite particles at pH 8.0. For an extreme high initial concentration of As(V) at 50 mg·L^−1^, MNPCs achieved an adsorption capacity under aerobic condition as high as 341.8 mg·g^−1^, which is the highest known to the authors' knowledge. Thus, this study showed that it is feasible to synthesize macro-scale MNPCs retaining similar adsorption capacity of magnetite particles with a diameter at around 1.0 nm. 

From the adsorption test results, MNPs and MNPCs exhibited different adsorption capacity for both As(III) and As(V). For the same concentration range (equilibrated concentration between 10 μg·L^−1^ and 25 mg·L^−1^), the adsorption capacity of MNPCs on As(V) was 1.16 to 2.25 times higher than those of MNPs under aerobic conditions, while the adsorption capacity of MNPCs on As (III) was 1.02 to 2.87 times higher than those of MNPs. This difference probably resulted from the effective distribution of MNPs on the surface of and the porous structure in the composite. Thus, higher adsorption capacity under aerobic conditions could be achieved by synthesizing a MNP composite. For MNP adsorption isotherms, unlike the phenomenon reported by other researchers that the adsorption curves of MNPs seemed to consist of two Langmuir isotherms [13,26], our results suggested there might be at least three plateaus in the curves for As(III) and As(V) and the phenomenon was more prominent for As(V). In addition, Langmuir and linear isotherms seemed to describe the adsorption behavior of MNPCs equally well, but an unrealistic q_max_, 1,981 mg·g^−1^, is obtained in fitting the Langmuir isotherm equation due to insufficient data points at higher concentration end.

Considering the potential adverse health effects of MNPs on humans, a recovery test was performed to compare these two adsorbents. By placing the serum bottles containing 0.1 g·L^−1^ of MNPs on a strong rare earth magnet for 30 min, about 602.5 ppb iron remained in the water indicating a 99.2% recovery. Even with such high recovery, for high concentration application, e.g., 25,000 μg·L^−^^1^, the adsorption capacities of MNP, 206.9 mg·g^−1^ and 168.8 mg·g^−1^ for As(V) and As(III), may still result in 172 μg·L^−^^1^and 139 μg·L^−^^1^of residual As(V) and As(III) in treated water, respectively. In other words, MNP-sorbed As will be less than 10 ppb if the initial concentration of As in the water is lower than 7,900 μg·L^−^^1^according to the fitted isotherm equation. For an immobilized approach as MNPCs, only about 8.5 ppb iron remained (>99.99 % recovery) causing no adverse health concerns at all. Considering the superior adsorption capacity of MNPCs under aerobic condition and the similar effectiveness on more toxic As(III) as MNPs under anaerobic conditions, MNPCs can offer not only a good option for point-of-use applications for As removal but also possess a niche in large-scale reactor type of treatments due to their lower residual concentration and higher compatibility with traditional sedimentation processes in current drinking water treatment.

## 3. Experimental Section

### 3.1. Synthesis of MNPs

Several methods have been reported to prepare MNPs [30,31]. Here, a coprecipitation method was modified and yielded good results. In short, FeCl_2_·4H_2_O (#2064-01, J.T. Baker, Chu-Bei City, Taiwan) and FeCl_3_·6H_2_O (#31232, Riedel-de Haën, Seelze, Germany) were mixed at a 1 to 2 ratio in a 100-mL amber glass bottle and NaOH was added to adjust the pH to 12. The amber glass bottle was then placed in a temperature-adjustable sonicator (Ultrasonic Cleaner DC600H, Delta New Instrument Co., New Taipei City, Taiwan) and kept at 80 °C for 30 min. Raw MNPs are thus synthesized and rinsed with deionized water (Millipore, Billerica, MA, USA) at least five times before undergoing vacuum drying in an anaerobic glove box (Coy Laboratory Products, Inc., Grass Lake, MI, USA). After drying, all MNPs were pooled into a bigger serum bottle and wrapped with aluminum foil for storage in the dark in an anaerobic chamber before use to avoid any oxidation caused by light or oxygen. 

In this study, a sol-gel method was modified to synthesize MNPCs using silane coupling agents for binding [32]. Briefly, hydrogen chloride (0.14 mL, 37.0%) was added into deionized water (1.2 mol or 21.6 g) to adjust the pH to 1.2 before adding isopropyl alcohol (0.3 mol). After thoroughly mixing, tetraethoxysilane (TEOS, #86578, Sigma-Aldrich, city, Taiwan, 0.3 mol) was added to start the condensation reaction. When the pH is less than 7.0, this silica gel tends to form chain-like or network-like structures [32]. Thus, it will be ideal to decorate MNPs on the surface to increase the SSA of MNPs in liquid phase. This reaction took 2.5 hours to finish and MNPs were added at the end of the second hour. These finished MNP-embedded silicate grains were then inter-linked by adding aminopropyltriethoxysilane (1 mL, APTES, #440140, Sigma-Aldrich) and vigorously mixing for 10 seconds. Then, this product was rinsed five times with deionized water, sealed in degassed water, and kept in the dark at 4 °C before use. However, due to the fire hazard of the reagents used in this synthesis method, the preparation is performed under aerobic conditions. 

### 3.2. Characterization of MNPs and MNPCs

Dried MNPs were characterized by three different instruments: *i.e.*, a transmission electron microscope (TEM; JEM-1400, JEOL, Tokyo, Japan), a powder x-ray diffractometer (XRD, X’pert Pro, Panalytical, Almelo, The Netherlands), and a Brunauer-Emmett-Teller (BET) surface area analyzer (ASAP 2020, Micromeritics, Norcross, GA, USA). The JEM-1400 TEM has a resolution at around 0.4 nm. MNPCs were only characterized by TEM.

### 3.3. Adsorption and Recovery Experiments

Stock solutions of arsenite and arsenate were prepared at 1,000 ppm and replaced weekly and monthly, respectively. Both solutions were kept in an anaerobic glove box before use. Right before the adsorption experiments, these stock solutions were diluted with a 0.01 M tris(hydroxymethylamino)methane buffer (Tris buffer) to 10, 50, 100, 250, 500, 1,000, 1,500, 2,500, 5,000, 10,000, and 25,000 ppb at a volume of 40 mL. For the adsorption test on MNPCs, 10, 100, 500, 1,000, 10,000, and 25,000 ppb were tested. With a Tris buffer, the pH is maintained at pH 8.0, a typical pH in most aquifer systems. The amount of adsorbent was 0.002 g of MNP in each 40-mL of test solution containing As (or 0.05 g·L^−1^ MNPs) or MNPCs with 0.002 g MNPs embedded in each 40-mL of test solution containing As (or 0.05 g·L^−1^ MNPCs, normalized to magnetite mass). All adsorption experiments were conducted for six hours at 30 °C. Anaerobic adsorption experiments were all performed in an anaerobic glove box (Coy Laboratory Products, Inc., Grass Lake, MI, USA) including water degassing, MNP reagent dilution, testing solution preparation, MNP transfer, mixing, magnetic separation, and supernatant sampling. For each test sample, 2 mL of supernatant were taken and transferred into a vial and sealed. Then, the samples underwent digestion and quantification using an inductively coupled plasma mass spectrometer (ICP-MS; ELAN DRC II, Perkin-Elmer, Inc., Waltham, MA, USA). To test the recovery efficiency of the MNPs and MNPCs, a recovery test is performed for both adsorbents. For this experiment, no As was added. MNPs were recovered by placing on a strong rare earth magnet for 30 min while the MNPC samples were left undisturbed on the bench top for 30 min. The supernatants were then transferred to new test tubes for digestion and quantification on an ICP-MS. All tests were conducted in triplicate with blanks.

### 3.4. Kinetic Study

A kinetic study was performed for MNPs to determine how fast the adsorption can reach equilibrium. Only environmentally-relevant concentrations were tested, *i.e.*, 10, 50, 100, and 500 μg·L^‑^^1^. The MNP amount in each 40-mL sample was 0.00130 g and the concentration was 0.0325 g·L^−1^. The adsorption experiments were sampled at 5, 10, 15, 30, 60, and 120 min. All samples were left on a rare earth magnet for at least 3 min and the supernatant were collected for digestion and ICP-MS quantification. The experiment was performed under anaerobic conditions at pH 8.0 to simulate typical As-containing groundwater. 

### 3.5 Sample Digestion

A volume of 2 mL of sample was placed into an acid-cleaned Teflon container with 2.5 mL of 70% nitric acid and 1.0 mL of hydrogen peroxide (30%, w/w). Then this container was put into a stainless container prior to inserting into a tightly sealed heater. Samples were heated to 300 °C for three hours and then cooled to room temperature. After digestion, the stainless containers were retrieved and rinsed with deionized water. The digested samples were transferred into a 10-mL volumetric flask. The volumes of digested samples were adjusted to a designated volume by adding deionized water before ICP-MS quantification.

### 3.6. ICP-MS Analysis

For ICP-MS analysis, radio frequency power was set at 1,050 W. Plasma gas flow, auxiliary gas flow, and nebulizer gas flow were set at 15 L·min^−1^, 0.85 L·min^−1^, and 0.75 L·min^−1^, respectively. Mass spectrometer pressure was at 2.0 × 10^−6^ Torr. Dwell time was 20 ms and scanning mode is peak hopping. Sweeps per reading were 250 and reading per replicate was four. The standard calibration had been performed and the R^2^ is equal to 1.0000 within the detection range. The method detection limits for As(III) and As(V) were less than 0.1 ppb.

## 4. Conclusions

This study demonstrated that ultrasmall MNPs are a good candidate material for efficient removal of As in water. It is feasible to synthesize a composite of larger size to retain its magnetic characteristics, adsorption capacity, and lower release of MNPs. MNPs not only showed high adsorption capacity, up to 168.8 mg·g^−1^ and 206.9 mg·g^−1^, for As(III) and As(V), respectively, but also exhibited fast kinetics. MNPCs synthesized in this study clear demonstrated it is feasible to increase the adsorption capacity of MNPs through their immobilization on a silica gel surface. These MNPCs are a very promising alternative to remove As from contaminated groundwater or drinking water due to their high compatibility with current water treatment technologies. 
